# Genome Sequence of *Mariprofundus* sp. Strain EBB-1, a Novel Marine Autotroph Isolated from an Iron-Sulfur Mineral

**DOI:** 10.1128/MRA.00995-19

**Published:** 2019-09-26

**Authors:** Areli Lopez, Daniel Albino, Senay Beraki, Sondra Broomell, Ricardo Canela, Theadora Dingmon, Sabrina Estrada, Marwin Fernandez, Pratixaben Savalia, Kenneth Nealson, David Emerson, Roman Barco, Benjamin J. Tully, Jan P. Amend

**Affiliations:** a Community College Cultivation Cohort, University of Southern California, Los Angeles, California, USA; b Department of Earth Sciences, University of Southern California, Los Angeles, California, USA; c Center for Dark Energy Biosphere Investigations, University of Southern California, Los Angeles, California, USA; d Department of Biological Sciences, University of Southern California, Los Angeles, California, USA; e Bigelow Laboratory for Ocean Sciences, East Boothbay, Maine, USA; Georgia Institute of Technology

## Abstract

Mariprofundus sp. strain EBB-1 was isolated from a pyrrhotite biofilm incubated in seawater from East Boothbay (ME, USA). Strain EBB-1 is an autotrophic member of the class Zetaproteobacteria with the ability to form iron oxide biominerals. Here, we present the 2.88-Mb genome sequence of EBB-1, which contains 2,656 putative protein-coding sequences.

## ANNOUNCEMENT

Members of the class *Zetaproteobacteria* are known for their ability to obtain energy from the oxidation of ferrous [Fe(II)] to ferric [Fe(III)] iron in microaerobic environments at circumneutral pH (1). *Zetaproteobacteria* species were initially associated with Fe(II)-rich deep-sea hydrothermal vents such as the Loihi Seamount (HI, USA), where the first isolate, Mariprofundus ferrooxydans, was discovered (1). However, *Zetaproteobacteria* species have also been found in shallow coastal marine environments, where they are thought to play roles in microbiologically influenced corrosion (2), rejuvenation of iron oxides in bioturbated sediments (3), cycling of iron in pelagic estuarine areas (4), and colonization of iron sulfides (5). Previously, pure cultures of iron-oxidizing Gammaproteobacteria and *Zetaproteobacteria* were established from *in situ* incubations of pyrrhotite, an iron-sulfur mineral, in a near-shore, marine environment of California (5, 6). Here, we present the genome sequence of *Mariprofundus* sp. strain EBB-1, isolated from the *in situ* incubation of pyrrhotite at the mouth of the Damariscotta River (i.e., saline water from the Gulf of Maine) (East Boothbay, ME, USA). EBB-1 was isolated from rusty material that accumulated as a biofilm on the surface of pyrrhotite. EBB-1 contributes to our understanding of the genomic potential of *Zetaproteobacteria* species and their potential ecological role with respect to iron, carbon, phosphorus, and nitrogen cycling in near-shore marine environments. EBB-1 is, to date, the only isolated strain representing the previously identified 16S rRNA-defined clade ZetaOTU14 in the class *Zetaproteobacteria*, order Mariprofundales, family Mariprofundaceae, genus *Mariprofundus* (7).

A sample of the rusty biofilm on pyrrhotite was incubated in artificial seawater medium (8) at room temperature in the presence of ferrous iron (FeCl_2_·4H_2_O) and under microoxic conditions, following the protocol by Barco et al. (5). A dilution-to-extinction culturing method (9) was applied for at least five transfers to isolate organisms under the described growth conditions. EBB-1 was selected for sequencing based on its capacity to grow in a medium under microoxic conditions with inorganic carbon as the sole carbon source and iron as the sole electron donor. EBB-1 genomic DNA was extracted using the FastDNA Spin soil kit (MP Biomedicals, Santa Ana, CA, USA), per the manufacturer’s protocol. Library construction and sequencing were performed at the Single Cell Genomics Center at the Bigelow Laboratory for Ocean Sciences following their published protocol (10). Library construction was performed using the Nextera XT (Illumina) reagents, following the manufacturer’s suggestions, except that DNA was purified using column cleanup kits (Qiagen) and size selected to 500 ± 50 bp using BluePippin (Sage Science, Beverly, MA, USA). DNA was sequenced using a NextSeq instrument (Illumina, USA) with 2 × 150-bp chemistry, generating a total of 7,248,670 raw paired-end reads. Trimmomatic version 0.32 (11) was used to trim the last 5 bp of each sequence, regions with low quality scores (Q < 15), and reads shorter than 36 bp, resulting in 7,092,091 quality-controlled sequences. These sequences were processed prior to assembly using a complexity filter threshold of 0.05, normalization with kmernorm version 1.05 (parameters *k* = 21; *t* = 30; *c* = 3; https://sourceforge.net/projects/kmernorm/). The resulting 723,807 high-quality paired-end sequences were then assembled using the SPAdes genome assembler version 3.9.0 (12), resulting in 63 high-quality contigs, with an *N*_50_ value of 84,398 bp and a maximum contig length of 200,406 bp. The assembled EBB-1 genome was 2,877,040 bp in length, with a GC content of 46.6%. Annotation was performed using the NCBI Prokaryotic Genome Annotation Pipeline (13, 14), resulting in 2,656 protein-coding genes, 3 rRNA genes (1 copy each of 5S, 16S, and 23S rRNA genes), and 38 tRNA genes. Preliminary assignment of EBB-1 to the genus *Mariprofundus* was determined using CheckM version 1.0.18 (15) and refined to a specific ZetaOTU by ZetaHunter (7).

As in other *Zetaproteobacteria* species, the EBB-1 genome contains genes indicative of the Calvin-Benson-Bassham cycle and high-affinity oxygen terminal oxidases (e.g., *cbb*_3_-type cytochrome oxidase), which are consistent with an autotrophic and microaerophilic lifestyle, respectively. Cyc2, the protein that has been proposed to be an iron oxidase in neutrophilic iron oxidizers (16), is also encoded in the EBB-1 genome. Additional analysis revealed that EBB-1 possessed genes encoding cellulase, sulfide:quinone oxidoreductase, chemotaxis, and flagellum biosynthesis.

### Data availability.

This whole-genome shotgun project has been deposited in DDBJ/ENA/GenBank under the accession number RCFQ00000000. The version associated with this submission is the first version, RCFQ01000000. The EBB-1 genome sequence has also been deposited in the JGI Integrated Microbial Genomes (IMG) and Microbiomes system under IMG identifier 2781125668. Raw reads have been deposited in the Sequence Read Archive under run number SRR9201317 and BioProject number PRJNA494876.
